# Advantages of using complement components in preventive and therapeutic vaccine strategies for infectious and non-infectious diseases

**DOI:** 10.1098/rsif.2025.0138

**Published:** 2025-07-02

**Authors:** Nur Hendri Wahyu Firdaus, Buddhadev Mallick, Kutty Selva Nandakumar, Akhilesh Kumar Shakya

**Affiliations:** ^1^ Department of Chemical Engineering, Texas Tech University, Lubbock, TX, USA; ^2^ Department of Zoology, Raniganj Girls College, Bardhaman, West Bengal, India; ^3^ Department of Medical Biochemistry and Biophysics, Karolinska Institute, Stockholm, Sweden; ^4^ Bioengineering, College of Engineering, Texas Tech University, Lubbock, TX, USA

**Keywords:** complement system, vaccine, adjuvants, autoimmunity, infections, cancer, neurological disorders

## Abstract

The complement system, a vital component of innate immunity, is indispensable to our immune defence mechanisms against microbial infections. Acting as a surveillance mechanism, it identifies and eliminates pathogens by activating several complement components and associated signalling pathways, which are also implicated in various diseases and disorders. Beyond its defensive role, the complement system has emerged as a promising target for vaccine development in therapeutic and preventive regimens, offering new vaccine strategies to combat non-infectious and infectious diseases. Activation of the complement pathways by various natural and synthetic adjuvants enhances protective immune responses, highlighting its utility in vaccine design. This approach could be useful for targeting autoimmune diseases, infectious diseases, cancer and neurological disorders.

## 1. Introduction

**Figure 1.**
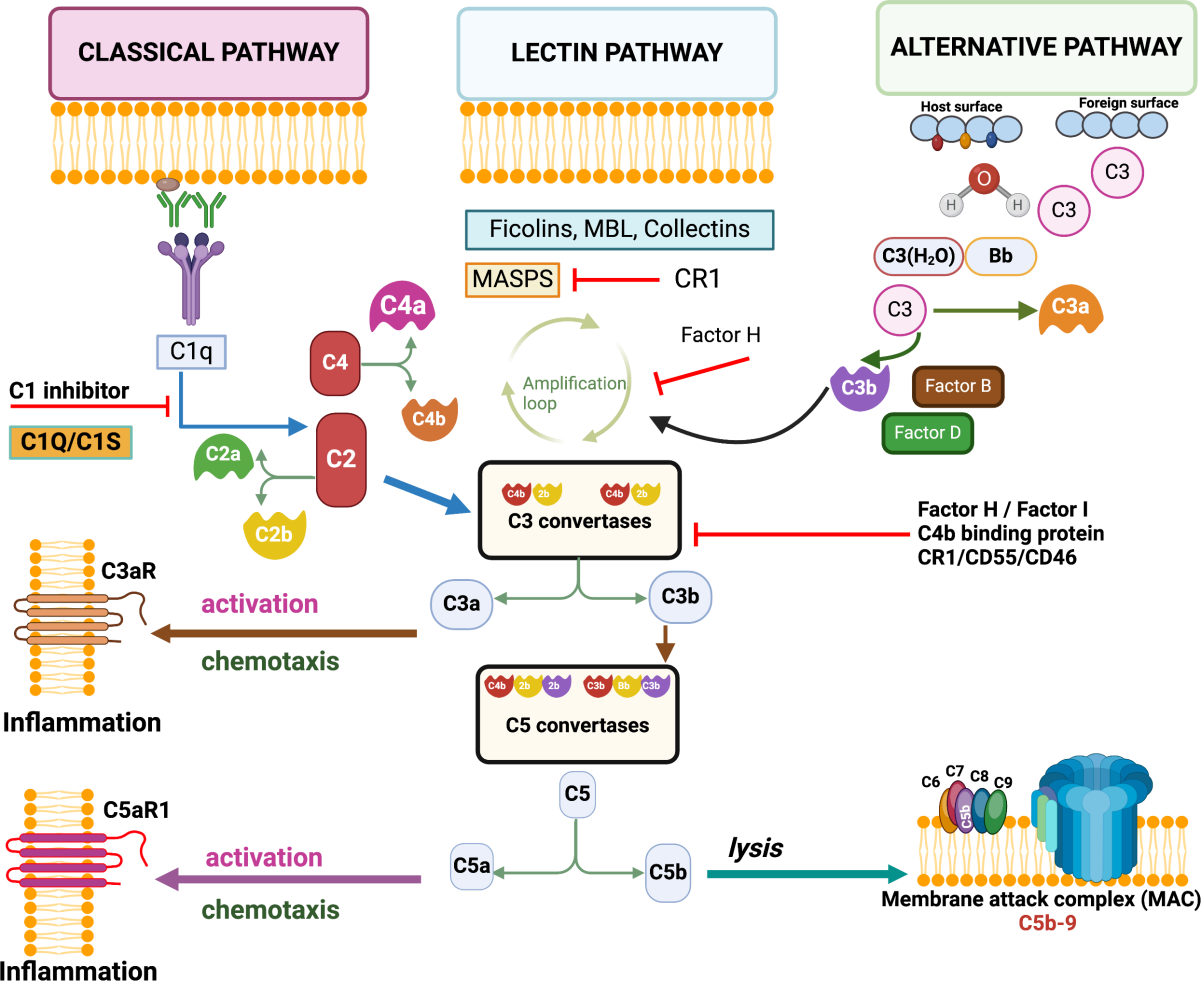
Overview of the complement system. Three different pathways of the complement activation—classical, alternative and lectin pathways. All three pathways are activated by different signals and merge into a common amplification point which involves the formation of C3 convertases cleaving C3 and leading to the formation of MAC on microbial surfaces to kill the microbes [8] .

Every day, we encounter several pathogens, and physical barriers like the skin, mucus and epithelial layers restrict their entry into our body. However, certain pathogens can cross these barriers and interact with the host immune system, primarily consisting of innate and adaptive components, to combat them. The innate immune system serves as the broader defence, killing and clearing pathogens from the body. A central part of this response is the complement system, which acts as a surveillance mechanism to detect and eliminate microbial threats [1–3]. Complement proteins are synthesized in the liver and released into the bloodstream as inactive precursors. However, extrahepatic synthesis of complement components was also documented [4]. These proteins become activated upon encountering microbes. Approximately 40 soluble and membrane-bound proteins, including cell surface receptors, are involved in the complement system, which can be activated through three distinct pathways to target and eliminate pathogens [5]. All three pathways happen on the pathogen surface, while the interaction of different plasma molecules initiates them [6]. First, the classical pathway is triggered by binding C1q to the antigen (present on the pathogen surface)–antibody (present in host plasma) complexes. C1q in host plasma binds to the Fc part of the antibody, leading to the activation of the C1 complex, which can also be activated by binding to pentraxins or bacterial surfaces. Subsequently, C1q activates C1r, which in turn activates C1s, promoting cleavage of C4 and C2 and the formation of C3 convertase (C4b2b), promoting further C3 cleavage. The alternative pathway is activated by polysaccharides, lipopolysaccharide (LPS) and aggregated IgA, leading to the formation of alternative pathway C3 convertase [C3(H_2_O)Bb], which cleaves C3. The third pathway is the mannose-binding lectin (MBL) pathway initiated by the binding of plasma MBL, ficolins and collectins to microbial surfaces, activating the MBL-associated serine proteases 1 and 2, leading to C3 convertase (C4b2b) formation [6,7]. Thus, all three pathways merge at the cleavage of C3 into C3a and C3b components by C3 convertases. Further down, C3 cleavage initiates a cascade of enzyme activities promoting cleavage of other complement proteins, including C5, with the generation of the membrane-attack complex (MAC) on the pathogen surface, the final product of all three pathways. The MAC creates a pore on the pathogen surface, which causes the free flow of solutes across the membrane, disrupting the membrane integrity of the pathogens and ultimately killing them [6,7]. The schematic of the complement cascade pathways is adapted from [8] (figure 1).

Under healthy conditions, the complement system typically does not attack our body cells due to the presence of plasma and membrane-bound regulators that control the system at various stages [9]. These regulators include C1 inhibitor, factor H and I, C4 binding protein, membrane cofactor (CD46), decay accelerating factor (CD55) and membrane inhibitor of reactive lysis (CD59) [10]. For example, CD55 inactivates the C3 and C5 convertase enzymes while CD46 regulates the deposition of C4b and C3b on the cells, and most importantly, CD59 restricts the MAC formation on the cell surface by blocking the polymerization of C5b, C6, C7, C8 and further incorporation of C9 component [11,12]. These regulatory proteins are generally expressed on almost every cell type and keep switching off the complement activity against the host cells. Microbial infection or immune deviation initiates the complement cascade. The involvement of the complement system in the pathogenesis of different diseases, including allergies, autoimmune diseases, cancer and neurological disorders, has already been comprehensively reviewed [13,14]. However, the vaccine potential of the complement components in prophylactic and therapeutic regimens for activation of the host immune system against infectious and non-infectious diseases has not been sufficiently discussed. Therefore, we mainly focused on and discussed complement-targeting vaccine efforts and the role of different adjuvants in activating the complement system.

## 2. Complement targeting vaccination efforts against microbes

Generally, a bacterial attack initiates a series of events in the complement system, forming MAC on the pathogen surface to kill it, in addition to the activation of other innate immune mechanisms like phagocytosis [15]. Pathogenic bacteria sometimes induce factors controlling the activation of the complement system, including the production of complement regulators (i.e. C4BP, factor H) and proteases cleaving the complement components [16]. Moreover, bacteria synthesize certain inhibitory molecules that block specific steps in the cascade of complement activation [17,18]. These inhibitory proteins are expressed on bacterial surfaces and act as immunogens. For example, bacterial pathogens evade complement attack by recruiting host complement regulators or secreting proteins that interfere with key components of the cascade, such as C3 and C5 convertases [19]. These molecules could be utilized to develop future vaccines against different bacterial infections. For instance, BipA, derived from *Bordetella pertussis*, protects against this infection in mice as a vaccine by reducing bacterial colonization in the lungs. Moreover, antibodies produced against BipA opsonized the bacteria more effectively [20]. Similarly, proteins such as FHA from *B. pertussis*, YadA from *Yersinia enterocolitica* and PspC from *Streptococcus pneumoniae* interact with complement regulators, allowing bacteria to evade complement attack [19]. Using immunoproteomics and TraDIS analysis, *B. pertussis* antigens, BipA, OmpP, OmpA2 and BamA were identified, which are essential for bacterial persistence and immunogenicity [21]. These findings reinforce the concept that antigens essential for bacterial survival that are immunogenic and surface-exposed are promising candidates for next-generation vaccines. Targeting such antigens, combined with optimized adjuvant formulations, could plausibly elicit balanced Th1/Th2/Th17 responses and enhance vaccine efficacy by facilitating bacterial clearance and reducing transmission. Incorporating these insights into vaccine design could address the limitations of current acellular pertussis vaccines, particularly their inability to prevent bacterial colonization and transmission.

The efficacy of vaccines targeting surface antigens from *Staphylococcus aureus* has been demonstrated in various studies. For instance, a combination of four antigens (IsdA, IsdB, SdrD and SdrE) has significantly induced protective immunity in mice by generating opsonophagocytic antibodies. Vaccinated mice exhibited a high level of protection against lethal challenges with clinical *S. aureus* isolates [22]. Building on this, immunoinformatics approaches recently facilitated the design of a multi-epitope vaccine targeting SdrD and SdrE proteins. This innovative vaccine, marked by high antigenicity with favourable safety profiles, was promising in eliciting both humoral and cellular immune responses while demonstrating broad population coverage as a cost-effective alternative to conventional vaccines [23]. A vaccine targeting SdrE prevented complement factor H (CFH) binding, increased C3b deposition, and enhanced bacterial clearance. SdrE enables *S. aureus* to evade complement attack by capturing CFH’s C-terminal tail, mimicking host cells to suppress C3 convertase activity and promote C3b degradation [24]

Similarly, the vaccine potential of *Pneumococcal* virulence proteins PspA and PspC has been explored due to their role in modulating the complement system. PspA inhibits both the classical and alternative complement pathways, preventing complement deposition and phagocytosis, thereby promoting *Streptococcus pneumoniae* virulence. PspC also modulates complement by binding to factor H, which inhibits the alternative pathway and impairs phagocytosis [25]. Intranasal administration of PspA expressed by *Lactobacillus casei* significantly protected mice from a pneumococcal challenge [26]. Although recombinant PspA and PspC combined with adjuvants induced high serum IgG levels, they did not reduce nasopharyngeal colonization of serotype 6B, highlighting the importance of IgG2c induction, infecting bacterial strain and immune response dynamics [27].

Vaccines targeting *Pseudomonas aeruginosa* containing its proteins have also demonstrated encouraging results. Immunization with OprF, OprI and flagellin proteins elicited strong antibody titres specific to these components, enabling an effective bacterial opsonization through complement activation and reducing lung damage and bacterial load in mice [28]. Recent strategies that enhance complement activity, such as the removal of cloaking antibodies protecting bacteria from serum-mediated killing and monoclonal antibody treatments blocking complement component decomposition, have shown promise in improving immune responses and therapeutic outcomes against *P. aeruginosa* [29]. Additionally, the use of ferritin nanoparticles to deliver OprI and PcrV antigens resulted in an adjuvant-free nano-vaccine (rePO-FN) that rapidly induced immunity and protected against *P. aeruginosa*-induced pneumonia in mice, offering a novel avenue for vaccine development [30]. This mechanism is linked to C3 activation by ferritin, an iron storage protein. Ferritin-induced iron overload upregulated C3, enhanced inflammatory responses and the production of reactive oxygen species (ROS), suggesting that ferritin nanoparticles could activate the complement system to boost immunogenicity [31].

The complement system has also been explored for developing future vaccines against mycobacterial infections. *Mycobacterium tuberculosis* activates all three complement pathways, leading to the deposition of C3 fragments such as C3b and iC3b on its surface [32]. Especially during the activation of the alternative pathway, mycobacterium binds to C3 and enhances phagocytosis of alveolar macrophages by engaging the complement receptor, CR3 [33]. CR3 is responsible for a large portion of complement-opsonized uptake; however, this pathway does not necessarily trigger bactericidal responses, potentially allowing intracellular survival of the pathogen [34,35]. In addition to its opsonic function, CR3 also facilitates non-opsonic phagocytosis by directly interacting with mycobacterial surface molecules, such as lipoarabinomannan (LAM) and phosphatidylinositol mannosides (PIMs)—through its lectin-like domain, independent of complement fragments [35]. This dual role of CR3 complicates its contribution to immune defence mechanisms, as non-opsonic uptake has been associated with immune evasion [34,35]. For example, oral vaccination against *Mycobacterium bovis* leads to increased levels of C3 in the serum. Strong expression of C3a promotes opsonophagocytic activity and rapid clearance of bacteria while inhibiting CR3-mediated opsonic or non-opsonic ways of mycobacterial uptake [34]. Recent studies further underscore the critical role of C3 in enhancing phagocytosis and clearing necrotic debris, which are crucial for immune responses and tissue recovery. Both C1q and C3 promote opsonization, facilitating the uptake of necrotic debris by neutrophils and macrophages. In the absence of C3, impaired phagocytosis hinders inflammation resolution and tissue regeneration. Therefore, complement dysfunction, particularly C3 deficiency, could compromise immune responses and delay recovery. Thus, C3’s involvement in immune responses and tissue repair highlights its potential as a target for enhancing vaccine efficacy, especially in individuals with complement deficiencies who might benefit from C3 supplementation to improve both immune functions and pathogen clearance [35].

## 3. Complement targeting preventive and therapeutic vaccine strategies against autoimmune diseases and cancer

**Figure 2.**
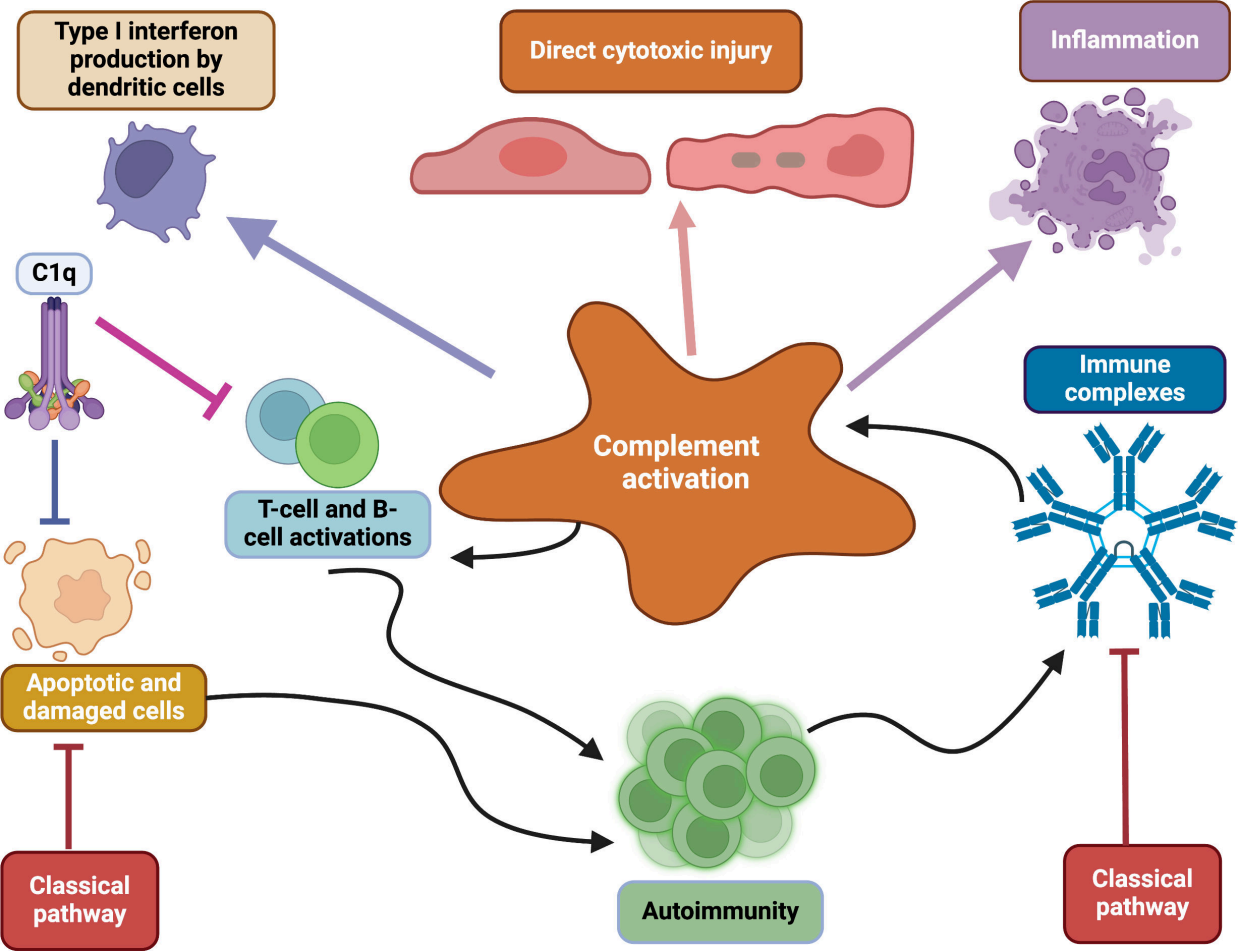
Dual role of the complement pathways in autoimmunity. The complement system prevents autoimmunity by inducing tolerance against evolutionarily conserved autoantigens or acts as a mediator of tissue damage and systemic inflammation by binding to autoantibodies. Moreover, complement deficiency can also lead to autoimmunity by altering the negative selection of autoreactive B cells and causing an inability to clear apoptotic cells and immune complexes [39].

The role of the complement system in autoimmunity is contradictory. On the one hand, activation of the complement system leads to tissue damage and contributes to the pathogenesis of autoimmune disorders. On the other hand, deficiencies in complement factors lead to autoimmunity. Therefore, a dual role for the complement system in autoimmune disorders is acknowledged. Involvement of the complement pathways and their components in the pathogenesis of autoimmune diseases such as systemic lupus erythematosus (SLE), rheumatoid arthritis (RA), multiple sclerosis (MS), vasculitis and Sjögren’s syndrome has been well established [36]. Complement activation by autoantibodies and immune complexes plays a significant role in promoting inflammation and tissue injury in SLE [37,38] (figure 2). Additionally, deficiencies in complement factors, particularly C1q and C4, are associated with more severe forms of disease, including earlier disease onset with the worst prognosis [40]. Recent studies have further clarified how C1q and C4 deficiencies impair apoptotic cell clearance, promoting autoimmunity and severe disease onset. Anti-C1q autoantibodies, associated with lupus nephritis, correlate well with disease activity and prognosis. Therefore, complement-targeting therapies offer promising approaches. Monoclonal antibodies like eculizumab (a C5 inhibitor) and its longer-acting successor ravulizumab inhibit C5 and reduce complement-mediated inflammation, while small molecules like avacopan provide alternatives to traditional treatments. Emerging RNA interference therapies and peptide inhibitors further highlight the growing potential of precision medicine in managing complement dysregulation [41].

Possibly, the waste-disposal hypothesis explains the role of the complement system in the pathogenesis of SLE [42]. This hypothesis explains the role of complement factors in the clearance of immune complexes (IC) and apoptotic cells from circulating blood and specific localized tissues, and the absence or malfunction of the complement system could lead to the deposition of IC and cell debris, provoking or initiating the disorder. This hypothesis consists of three steps; the first step involves the failure of autoantigen clearance from the tissues, and the second step deals with the uptake of autoantigens by the immature dendritic cells in the presence of inflammatory cytokines, leading to the maturation of DCs into professional antigen-presenting cells (APCs). This step also comprises autoantigen presentation to autoreactive T lymphocytes [43]. Finally, the third step is T- and B-cell maturation and secretion of autoantibodies [44]. The severity of SLE depends on complement factors present in the classical pathway. Generally, C1q and C4 deficiencies lead to severe SLE symptoms early in life, while C2 deficiency is associated with milder symptoms during the later phase of life [45]. In addition to the classical pathway, the alternative and lectin pathways also contribute to SLE pathogenesis [46]. Complement components such as cell-bound complement activation products (CB-CAPs) are emerging as reliable biomarkers for diagnosis and prognosis [47]. Therapeutic advances, including complement inhibitors like CRIg/FH, show great promise in managing severe forms of the disease, like lupus nephritis [48].

Similar to SLE, the role of complement is also ambiguous in RA. For instance, preventive immunization of C3 or factor B deficient mice with bovine CII suppressed or completely ameliorated arthritis development [49]. Blocking C5 activation using anti-C5 monoclonal antibodies prevented arthritis onset in susceptible mice and also improved the ongoing clinical symptoms [50]. The anaphylatoxin C5a, a cleaved product of C5, interacts with its receptors C5aR (CD88) and C5L2 (GPR77) expressed on immune and non-immune cells and promotes pro-inflammatory activities [51,52]. Therefore, attempts were made to develop a preventive and therapeutic vaccine to induce C5a-neutralizing antibodies without compromising on C5/C5b activity by breaking tolerance to the endogenous C5a. Interestingly, preventive and therapeutic vaccination with a fusion protein of C5a and maltose-binding protein induced C5a-neutralizing antibodies, which significantly reduced the incidence and severity of joint inflammation in three models of experimental arthritis [53]. Subsequently, an anti-C5a preventive vaccine with an unnatural amino acid p-nitrophenylalanine (4NPA) replacing a tyrosine residue, especially at position 35, in the murine C5a molecule, was found to induce loss of tolerance towards the endogenous protein, which partly protected mice against arthritis development while strongly reducing the disease severity [54]. A recent study reported the previously unidentified role of lymphocyte-derived granzyme K in activating the complement cascade in arthritis [55]. Therefore, activation of the complement system is possibly involved in the onset of disease, and deficiency of these components or blocking their activities might also correlate with RA development [56,57]. Several mechanisms have been developed to explain the involvement of the complement system in RA pathogenesis. Generally, patients with RA are diagnosed with elevated levels of systemic ICs and rheumatic factors, which activate the classical pathway of the complement system [58]. Moreover, the alternative pathway is also activated in RA, as observed with decreased levels of factor B and properdin, and elevated concentrations of Ba in the synovial fluid [59]. The involvement of the lectin pathway is also confirmed in the establishment of RA by changing IgG glycosylation that causes enhanced binding of MBL proteins, activating the complement cascade [60]. Recent studies identify local tissue priming as a key factor in RA progression. Repeated inflammatory stimuli activate synovial fibroblasts (SFs) and enhance their metabolic activity, migration and invasiveness, sustaining joint inflammation and damage. C3 is upregulated in the SFs during inflammation and plays a central role in this process. Single-cell RNA sequencing reveals an elevated C3 expression in specific SF subsets within leukocyte-rich RA synovial tissue, linking it to higher disease activity. These findings suggest that targeting C3-mediated mechanisms could reduce inflammation and structural damage in RA without causing systemic immunosuppression [61].

A possible but not definite role of the complement system has also been demonstrated in systemic sclerosis (SSc). Elevated levels of complement fragments were observed in patients diagnosed with cutaneous SSc compared with controls [62]. However, Hudson *et al.* [63] did not observe any considerable differences in the clinical presentation of disease between healthy and 321 SSc patients having low levels of complement factors. Besides, the role of the complement system was also demonstrated in other autoimmune disorders like type 1 diabetes, vasculitis, dermatomyositis and antiphospholipid syndrome [64,65]. So far, few therapeutics (e.g. eculizumab and plasma C1 protease inhibitor) have been approved to correct these autoimmune disorders. Eculizumab is the first approved antagonist (C5 inhibitor) that binds specifically to C5, blocking its cleavage [66]. Although it was developed to treat SLE, RA and dermatomyositis, initially, it was approved for treating paroxysmal nocturnal haemoglobinuria (PNH). PNH is a blood-related disorder characterized by symptoms like haemolytic anaemia, bone marrow failure and thrombosis [67]. Recent advances have expanded the scope of complement-targeting therapeutics. Ravulizumab, approved in 2018 by the US Food and Drug Administration (FDA), is a longer-acting derivative of Eculizumab for treating PNH and atypical haemolytic uremic syndrome (aHUS), offering improved treatment options [68]. Similarly, Pegcetacoplan, a C3 inhibitor, efficacy was demonstrated in PNH, and its use is under investigation for other complement-mediated conditions [69]. These developments underscore the growing importance of complement modulation as a therapeutic strategy in autoimmune disorders.

## 4. Complement targeting therapeutic vaccine strategies against neurological diseases

The complement system, a key component of the innate immune response, plays a crucial role in the pathogenesis of several neurological diseases as well, by mediating inflammation, synaptic pruning and neuronal damage (table 1). Dysregulation of complement activation has been implicated in the progression of disorders such as Alzheimer’s disease (AD) [74], MS [75], Parkinson’s disease (PD) [76,77] and amyotrophic lateral sclerosis (ALS) [78,79]. Recent studies suggest targeting the complement system could offer a promising therapeutic approach in the form of a therapeutic vaccine to mitigate neuroinflammation and protect neuronal function [71,72,80]. This strategy includes modulating complement components like C1q, C3, C5 and CR3 to regulate inflammatory processes, reduce amyloid pathology and prevent excessive synaptic pruning. This section explores the potential of complement-targeting therapeutic vaccine strategies for the treatment of neurological diseases, with a focus on their roles in modulating disease progression and offering new avenues for therapeutic intervention.

**Table 1.** Preclinical preventive and therapeutic vaccination studies targeting the complement system in neurological diseases.

neurological diseases	immunized antigens	targeted complement components	output
Alzheimer’s disease [70]	amyloid beta	C3	C3aR antagonists ameliorated amyloid plaque load, microgliosis and improve cognitive function by blocking complement activation and neuroinflammation.
multiple sclerosis [71]	intrathecal IgM and IgG synthesis	C3a, C4a, Ba, Bb	increased levels of complement activation products (C3a, C4a, Ba and Bb) were found in the CSF of MS patients, especially those with intrathecal IgM synthesis. Elevated complement levels correlated with higher disease severity and neuroaxonal damage.
Parkinson’s disease [72]	α-synuclein preformed fibrils	C3	α-synuclein activates the complement system in PD, increasing C3 levels in astrocytes, exacerbating neurodegeneration through the C3/C3aR pathway and affecting GSK3β activity. Targeting C3 could offer a potential therapeutic approach.
amyotrophic lateral sclerosis [73]	MSC-derived small extracellular vesicles	C1q	EVs reduced C1q overactivation, alleviated neuroinflammation, improved motor functions and extended survival in SOD1G93A ALS mice.

ALS, amyotrophic lateral sclerosis; CSF, cerebrospinal fluid; EV, extracellular vesicles; GSK3β, glycogen synthase kinase 3 beta; MS, Multiple sclerosis; MSC, mesenchymal stem cell; PD, Parkinson’s disease.

### 4.1. Alzheimer’s disease

AD is a progressive neurodegenerative global disorder and the leading cause of dementia, characterized by memory deficits and cognitive decline, having hallmark pathological features such as amyloid plaques and neurofibrillary tangles [74]. Many therapeutic approaches have been explored to mitigate AD progression, including targeting the complement system with a focus on inhibiting excessive synaptic pruning and reducing inflammation. Blocking C1q, C3 or CR3 protects synapses and improves cognitive outcomes in mouse models. Targeting the C5a-C5aR1 pathway with antagonists (e.g. PMX205) reduces amyloid pathology, glial activation and memory deficits while maintaining the protective roles of C1q and C3. Complement inhibitors like Crry and modulation of microglial pathways (e.g. SIRPα) also help to prevent over-pruning, providing a balanced approach to mitigate AD progression [80]. While complement-based therapies offer new hope, challenges remain in AD treatment, including insufficient specificity and a lack of biomarkers to guide intervention in amyloid β-based therapies [81]. Further investigations into the complement-mediated mechanisms, along with the development of biomarkers and combination therapies, could pave the way for more refined, personalized strategies to address AD’s complex pathology.

### 4.2. Multiple sclerosis

MS is a chronic autoimmune disorder of the central nervous system (CNS) characterized by inflammatory demyelination and neurodegeneration. Dysregulation of the complement system plays a pivotal role in MS pathology, particularly in progressive disease stages, through mechanisms such as complement-mediated myelin phagocytosis, axonal injury and chronic microglial activation [75]. Complement-targeting approaches in MS focus on modulating complement activity to balance its protective and pathological roles. These include pharmacological inhibitors, such as the anti-C5 antibody eculizumab, which aim to prevent harmful complement activation by targeting and inhibiting the C5 complement factor, support debris clearance for remyelination and reduce interactions between complement and known risk factors like Epstein–Barr virus (EBV). Insights from knockout studies in experimental autoimmune encephalomyelitis (EAE) models guide pathway-specific targeting, while advanced CNS delivery systems aim to improve therapeutic efficacy [75]. Recent studies provide additional support for the role of complement activation in MS, identifying elevated levels of activation products (e.g. C3a, C4a, Ba and Bb) in the cerebrospinal fluid (CSF) of patients with MS. This activation correlates with the disease severity and neuroaxonal damage, emphasizing complement-targeting therapy’s potential to mitigate progressive disease [71]. Recent findings by Saez-Calveras and Stuve [75] revealed the dynamic cellular interactions occurring in MS lesions, showing an active lesion expansion and the emergence of disease-associated glia that dynamically resolve throughout the disease course. Together, these insights refine the understanding of MS pathology and guide the development of targeted interventions. In addition to targeting the complement system, therapies focused on EBV, a known risk factor in MS, are aimed at reducing inflammation and depleting infected B cells. CD20-specific therapies like ocrelizumab and ofatumumab effectively lower relapse rates and lesion formation, while teriflunomide suppresses inflammation and EBV-induced lymphoproliferation [82].

### 4.3. Parkinson’s disease

PD is the second most common neurodegenerative disorder, with its prevalence expected to double in the next 30 years. It is characterized by progressive motor and non-motor symptoms, driven by α-synuclein aggregation, mitochondrial and lysosomal dysfunctions and inflammatory processes. Despite advances in diagnostic criteria, biomarkers and identification of genetic subtypes, early diagnosis remains a significant challenge. Emerging evidence highlights the role of the complement system in PD pathogenesis, with maladaptive immune and inflammatory responses potentially originating in the gut, accelerating disease progression [76,77]. Recently, Chi *et al*. [72] expanded this understanding by demonstrating the role of C3, activated through the C3/C3aR pathway in astrocytes, in exacerbating neuronal apoptosis and α-synuclein pathology in PD. This highlights a second pivotal component in the complement activation, besides C1q, offering new therapeutic targets against disease development. Complement levels in the serum, including C1q, differ significantly between patients with PD, healthy controls and individuals with 4R-tauopathies, suggesting its potential utility for disease differentiation [83]. Experimental models further illustrate C1q’s dual roles, not only in facilitating extracellular debris clearance through microglial activity [84], but also contributing to neurodegeneration when interacting with α-synuclein aggregates [85]. Recent preventive vaccine strategies targeting α-synuclein aggregation have also explored complement-modulating adjuvants, including those that could modify C1q activity [86]. Furthermore, complement-modulating vaccines might also benefit from incorporating strategies to regulate C3, which could potentially enhance the therapeutic efficacy by targeting both C1q and C3 pathways in PD [72].

### 4.4. Amyotrophic lateral sclerosis

ALS is a fatal neurodegenerative disorder of the CNS, characterized by progressive loss of motor neurons, leading to muscle weakness, atrophy and eventual respiratory failure [78,79]. While the exact pathogenesis of ALS remains unclear, mounting evidence highlights the role of neuroinflammation in its progression, with the complement system emerging as a significant player through its involvement in synaptic pruning, clearance of cellular debris and modulation of inflammation. Dysregulation of this system may exacerbate neuronal damage and contribute to ALS progression [78]. Recent proteomic and transcriptomic analyses revealed dysregulation of complement pathways in SOD1G93A ALS mice. The mesenchymal stem cell-derived small extracellular vesicles (sEV) treatment downregulated the complement-coagulation cascade, NF-ĸB signalling and the key inflammatory mediators like C3. This alleviated neuroinflammation, delayed motor decline and prolonged survival, highlighting sEVs as a promising multi-targeted ALS therapy [73]. Another study evaluated the safety and efficacy of ravulizumab, a C5 inhibitor, in slowing functional decline in ALS. This phase 3, double-blind, placebo-controlled trial involved 382 participants randomized to receive ravulizumab or a placebo over 42 weeks with standard-of-care treatments. Results showed no significant difference in the revised amyotrophic lateral sclerosis functional rating scale (ALSFRS-R) or the combined assessment of function and survival (CAFS) scores, and the trial was terminated early. Safety profiles were similar, with treatment-associated adverse events in 80% of the ravulizumab group compared with 85% of the placebo group. These findings underscore the complexity of complement activation in ALS and highlight the need for alternative therapeutic strategies to address disease progression and survival [87].

## 5. Complement factors targeting preventive vaccination efforts against viruses

The complement system plays a crucial role in antiviral immunity by bridging innate and adaptive responses (table 2). Immune complexes, formed by antibody-bound antigens, engage complement and complement receptors to enhance viral clearance and modulate immune responses. These interactions influence preventive vaccine efficacy by shaping the quality and magnitude of adaptive immunity. Given this, complement-targeting strategies have gained interest in vaccine development. This section examines how complement factors contribute to vaccination efforts against various viral diseases.

**Table 2.** Preclinical preventive vaccination studies against different viruses targeting the complement system or their components.

viruses	immunized antigens	targeted complement components	output
dengue virus [88]	OmpH from *Y. enterocolitica*	C5aR	vaccination provided protection against all serotypes of dengue virus by activating Th2-type cellular responses via involvement of the complement system.
influenza virus [89–91]	M2e universal influenza antigen	C3a	activated influenza virus-specific CD4^+^ and CD8^+^T cells generated long-term memory responses.
human cytomegalovirus [92]	a combination of envelope pentamer complex, glycoprotein B and phosphoprotein 65 antigens	not specified	induced complement-dependent and complement-independent antibodies contributed to immunity against HCMV infection.
herpes simplex virus 2 [93]	HSV-2 glycoprotein C (gC2)+gD2	C3b	antibodies produced in mice immunized with gC2 prevented the interactions between gC2 and C3b and protected against HSV-2 infection.
human immunodeficiency virus [94]	rILYd4 toxin	complement-based lysis	the rILYd4 toxin interacted with human CD59 and protected against virus by preventing its escape from complement-based lysis.

HCMV, human cytomegalovirus; HSV-2, herpes simplex virus 2; rILYd4, human CD59 inhibitor.

### 5.1. Dengue virus

Dengue virus (DENV) infection is a health burden for billions of people around the world. Many strategies and approaches are underway to develop an effective vaccination strategy against all dengue virus serotypes, which also includes targeting the manifold (M) cells expressing the C5a receptor (C5aR). A study involving C5aR as a target was explored in inducing immune responses by using the major outer membrane protein, OmpH, derived from *Y. enterocolitica* [95] and further investigated for its role as a ligand [88]. OmpH ligand was conjugated to EDIII protein (an antigenic protein of dengue virus) for activating mucosal and systemic immune responses after oral immunization of mice. OmpH-mediated delivery targeting M cells induced activation of Th2-mediated cellular immune responses, which protected mice vaccinated with OmpH-EDIII protein against all serotypes of dengue virus [88]. In parallel, advances in tetravalent vaccine development have further contributed to achieving broad serotype protection. The TAK-003 vaccine, evaluated in a large-scale, multi-country clinical trial, demonstrated long-term efficacy and safety in preventing symptomatic dengue disease across all serotypes in previously exposed individuals (NCT02747927). This vaccine showed 84.1% efficacy in preventing hospitalizations and was effective against DENV-1 and DENV-2 infections in baseline seronegative participants [96].

### 5.2. Influenza virus

Involvement of the complement system has been demonstrated in influenza virus infection, as the presence of anaphylatoxins, C3a and C5a, was observed in the upper and lower airway mucosal surfaces of influenza-infected patients [97]. These plasma components of the complement system help to reduce the virus burden in the early phase of infection by clearing pathogens by neutralization, opsonization, phagocytosis or lysis [98]. For instance, in influenza infection, the host immune response involves activation of the complement system along with the generation of antibodies against the membrane proteins like haemagglutinin (HA) and neuraminidase (NA). Influenza virus-specific antibodies bind to the infected cells and promote lysis of virus-infected cells through the involvement of C3a and C5a in a process widely recognized as complement-dependent lysis [99]. The importance of C3a was recognized in influenza virus infection, while using C3a-deficient mice, which showed a delay in virus clearance along with a high level of viral load in the lungs [89]. Additionally, C3a also contributes by activating influenza virus-specific CD4^+^ and CD8^+^ T cells, creating a long-term immune response [89,90]. A recent study demonstrated the role of C3 in activating adaptive immune responses while developing universal M2 ion channel extracellular domain (M2e)-based influenza vaccines [91]. Recent advances in influenza vaccine research have focused on overcoming the limitations of strain-specific immunity by targeting conserved viral antigens. A novel mRNA-based multi-epitope vaccine has shown promising results in preclinical studies. This vaccine encodes conserved proteins such as M2e and epitopes from haemagglutinin, eliciting robust humoral and cellular immune responses, including activation of CD4^+^ and CD8^+^ T cells. Vaccinated mice exhibited significantly reduced viral loads, lung damage and pro-inflammatory cytokine levels after the virus challenge, demonstrating enhanced cross-protection against H1N1 and B influenza viruses [100].

### 5.3. Human cytomegalovirus and herpes simplex virus

The complement system involvement was also noted against human cytomegalovirus (HCMV), which generally causes disease in transplant recipients and newborns. A vaccine based on the envelope pentamer complex (PC), glycoprotein B and phosphoprotein 65 antigens induced robust HCMV-specific humoral and cellular responses. Mice immunized with this construct generated complement-dependent and independent antibodies, significantly contributing to the immunity against HCMV [92]. The proteins involved in immune evasion from the complement attack have also been used to design a vaccine against herpes simplex virus 2 (HSV-2). For instance, the vaccine potency of HSV-2 glycoprotein C (gC2), a protein known for complement evasion, has been tested along with gD2 antigen in mice and guinea pigs to determine whether the addition of an evasion molecule in gD2 can improve the protection against HSV-2 infection. Antibodies produced in immunized mice with gC2 were able to prevent the interactions between gC2 and C3b. Moreover, the passive transfer of gC2 antibodies protected complement-sufficient mice against HSV-2 challenge compared with C3-deficient mice, which had negligible protection. A high titre of neutralizing antibodies was also detected in mice immunized with the gC2 protein. Moreover, mice immunized with gC2 and gD2 had protection of their dorsal root ganglion and developed a high level of CD4^+^ T-cell response. Interestingly, a low frequency of recurrent vaginal shedding of virus was also noticed in guinea pigs immunized with gC2 and gD2 proteins [93]. Building on these insights of HSV-2 immune evasion and vaccine strategies, recent advances highlighted novel therapeutic and prophylactic approaches. One such development involves the role of peptidylarginine deiminase-3 (PAD3), a key enzyme in protein modification, which HSV-2 exploits to enhance its replication. Inhibiting PAD3 with a specific inhibitor, CAY10727, markedly reduced viral replication and protein production, suggesting PAD3 is a potential therapeutic target for HSV-2 infection, especially those resistant to existing antiviral drugs [101]. Additionally, a trivalent HSV-2 mRNA-lipid nanoparticle (LNP) vaccine (gC2, gD2 and gE2) has shown a remarkable efficacy in preclinical models, providing robust protection against HSV-1 infection in the lip and genitals. This vaccine has not only induced a potent neutralizing antibody but also elicited unexpectedly strong CD8^+^ T-cell responses, comparable with or exceeding those generated by an HSV-1-specific mRNA vaccine. Both vaccines effectively prevented clinical disease, mortality and viral spread to critical nerve ganglia, underscoring the potential of the HSV-2 vaccine as a universal prophylactic solution for both HSV-1 and HSV-2 infections [102].

### 5.4. Human immunodeficiency virus

Generally, in human immunodeficiency virus (HIV) infection, the host immune system produces antibodies against multiple epitopes of the viral antigens and some of them are neutralizing antibodies. These antibodies contribute to antiviral immunity in various ways, which include direct neutralization of virus particles, initiation of complement-mediated lysis along with non-neutralizing antibodies, participation in phagocytosis, opsonization and antibody-dependent cellular cytotoxicity [103]. These processes help in clearing virions or killing the infected cells, involving complement activation. Therefore, neutralizing antibodies were further modified to enhance the involvement of the complement system in humoral immunity against HIV infection. To escape complement-mediated clearance, the HIV envelope possesses certain complement inhibitors like CD55 and CD59 [104]. This could be the explanation why, generally, patients with HIV do not show remission even in the presence of neutralizing antibodies. Hence, strategies were developed to utilize these molecules as a potential target for future HIV vaccination. For instance, a recombinant fourth form of bacterial toxin (rILYd4) was evaluated as a vaccine against HIV [94,105]. This toxin binds with high specificity and affinity to human CD59 and prevents the escape of the virus from complement-mediated lysis [94]. To develop an effective vaccine for HIV, nanoparticles (NPs) were also investigated to enhance humoral immunity by promoting complement-mediated recognition. For example, the efficacy of glycosylated HIV antigens, gp120 mini protein and a large protein, either arrayed on NPs or in free forms, was compared in mice. Unlike free forms, antigens-decorated NPs were found to be effective in promoting immune responses by activating the complement system [106]. Recent advances in HIV vaccine research have focused on overcoming the challenges of eliciting broadly neutralizing antibodies (bnAbs) [107] by introducing a germline-targeting approach using engineered epitope scaffolds and nanoparticles to elicit 10E8-class bnAb precursors targeting the recessed gp41 epitope. This strategy successfully triggered bnAb-precursor responses in stringent animal models, including mice and rhesus macaques, and demonstrated the potential of mRNA-encoded NPs in enhancing immune responses. Similarly, Saunders *et al.* [108] developed a CD4-binding site (CD4bs)-targeting immunogen that activated bnAb precursors with structural and genetic features of CD4-mimicking bnAbs in macaques. This study represents a critical step in initiating B cells capable of producing CD4bs-bnAbs, addressing a key obstacle in HIV vaccine development. Together, these findings highlight innovative approaches to designing immunogens that effectively prime bnAb responses.

## 6. Complement targeting vaccines against parasite-based infections

The indirect role of complement components was assessed in vaccines against parasitic infections. For example, in malaria, C5a was targeted in the development of a whole-killed parasite vaccine at the blood stage of infection. Mice immunized with red blood cell (RBC) lysate and infected with *Plasmodium yoelii* increased C5a levels in the blood. The role of C5aR was confirmed in this study using C5aR-deficient mice, which had poor protection, associated with low-level activation and differentiation of CD4^+^ T cells. Similarly, administering C5aR antagonists in B-cell-deficient mice confirmed the earlier observation of reduced protection. Moreover, defective CD4^+^ T-cell responses in C5aR-deficient mice were associated with poor activation of dendritic cells and expansion of CD4^+^CD25^+^foxp3^+^ Treg cells [109]. Recently, the induction of complement-fixing antibodies post-immunization with plasmodium antigens was reported to be an important aspect of gaining immunity against malaria. For instance, in a human study, the *Plasmodium falciparum* sporozoites (PfSPZ) vaccine induced anti-PfCSP IgG and IgM antibodies. The complement-fixing activities of IgM antibodies were also investigated by analysing the inhibitory activities on sporozoite invasion of *P. falciparum* in hepatocytes [110]. Generally, complement-fixing antibodies are antigen-specific, which promote the deposition of complement proteins on merozoites or sporozoites to inhibit their erythrocyte invasion. This mechanism includes C1q deposition and activation of the classical complement pathway. The complement-dependent erythrocyte invasion inhibition is the major activity of complement-fixing antibodies [111]. Inhibition through complement-dependent antibodies can induce protection against clinical malaria and high-density parasitemia in children [111]. A leading malarial vaccine, RTS, S/AS01, is based on the circumsporozoite (CSP) protein of sporozoites, which showed protection against malaria in 1- to 4-year-old children. CSP-based vaccine-induced complement-fixing anti-CSP antibodies, mostly including IgG1, IgG2, IgG3 and IgM subtypes. Anti-CSP antibodies interacted with complement factors and conferred protection against malaria. These complement-fixing antibodies are short-lived and mostly recognize the central repeat and C-terminal parts of the CSP. Interestingly, the level of these complement-fixing antibodies decreases with age, as the decline of these antibodies was observed within months post-vaccination [112]. The role of complement-fixing antibodies was also characterized in another study based on *Plasmodium vivax* merozoite surface protein 3α (PvMSP3α) [113]. In this study, complement-fixing antibodies comprising IgG1, IgG3 and IgM were elevated against the central region of PvMSP3α. These antibodies mediated C1q-fixation during *P. vivax* infection. A significant difference was not found in complement-fixing antibodies between different age groups of people. However, the level of these antibodies increased post 7 days of acute infection and declined in the fourth week [113]. More studies are needed to understand the detailed role of complement-fixing antibodies and their interactions with the complement system to elicit effective immunity against malaria.

**Figure 3.**
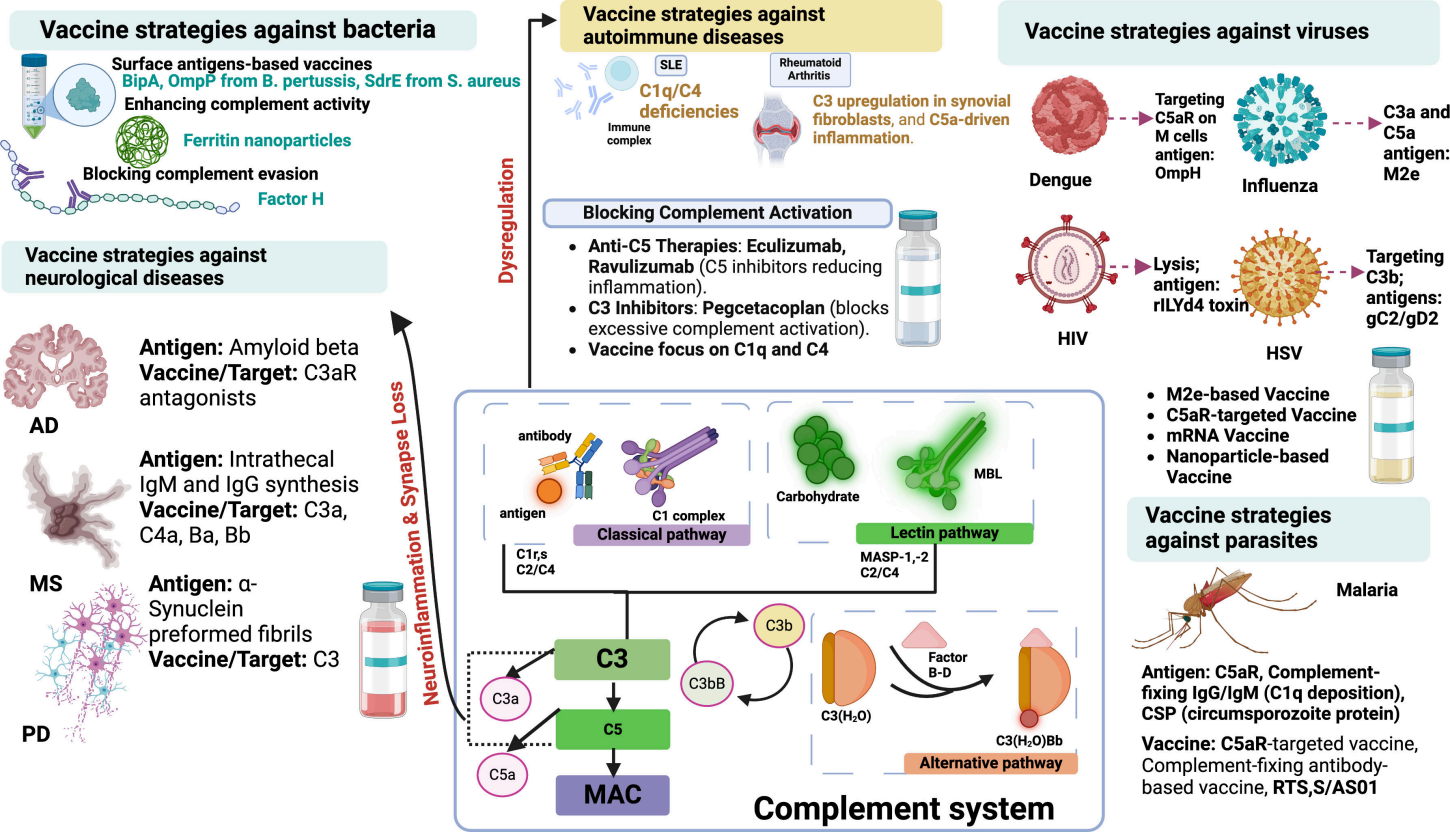
Overview of vaccination strategies targeting the complement system against microbes, including bacteria, viruses, neurological diseases, autoimmune diseases and parasites.

Recent advances in malaria vaccine research underscore the importance of humoral immunity in achieving durable protection. The R21/MM vaccine has demonstrated high efficacy (77% at 1 year) and robust anti-NANP antibody responses in African children [114]. Based on this, a subsequent study revealed that a booster dose administered 1 year after the primary immunization maintained a higher efficacy, with elevated antibody titres correlating strongly with protection [115]. These findings highlight the potential of antibody-mediated immunity, including complement interactions, in sustaining the host defence mechanisms against malarial infection. Together, these approaches contribute to a broader vaccine development strategy, as depicted in figure 3.

## 7. Adjuvants for activation of the complement system

To induce adaptive immunity against microbes, the potential of the complement system has been explored in different preventive vaccination studies using various adjuvants (table 3). The following section summarizes different natural and synthetic materials explored for activating the complement system to enhance antigen-specific immune responses.

**Table 3.** A list of different adjuvants demonstrated in vaccination studies targeting the complement system.

adjuvant	antigen(s)	targeted complement components	output
chitosan microparticles [116]	anthrax antigen	C3b	free amino groups containing chitosan microparticles interacted with C3b and stabilize it for a longer period of time thus sustaining complement activation
gamma Inulin [117,118]	—	C3	gamma inulin activated the complement system by increasing deposition of C3 on APCs (macrophages), leading to T-cell activation
beta glucans [119]	—	CR3	beta glucans induced production of complement-dependent and complement-independent antibodies, which significantly contributed to immunity against HCMV infection
zymosan [120]	HIV-1 DNA	complement components	zymosan induced high levels of antibody responses and HIV-specific delayed type hypersensitivity in immunized mice
alum [121]	—	C3a and C5a	alum induced production of anaphylatoxins (C3a and C5a), formation of MAC and deposition of C3 cleavage components
polymethoxylated (CH_3_O-NPs) and polyhydroxylated (OH-NPs) NPs [122]	—	C3a	hydroxylated NPs targeted lymph nodes promoting DC maturation and activation of CD8+ T-cell memory
flagellin [123]	vaccinia virus antigens in fusion forms like flaggelin-L1R and flaggelin-B5R	complement components	immunization with fusion proteins protected mice against vaccinia virus by activating the complement system

APC, antigen presenting cells; DC, dendritic cells; HCMV, human cytomegalovirus; HIV, human immunodeficiency virus; MAC, membrane attack complex; NP, nanoparticle.

### 7.1. Chitosan

Chitosan is a natural polymer, and it has been explored as an adjuvant in different vaccination studies [124]. Recently, chitosan’s role as an activator for the complement system to boost immune responses has also been demonstrated. Chitosan microparticles (CS-NH_2_ MPs) were utilized as a vaccine adjuvant for activation of the complement system due to the availability of amino groups present on the microparticle surface. The capacity of free amino groups was compared with cross-linked (CS-CL MPs) chitosan microparticles containing anthrax antigens. CS-NH_2_ MPs were observed to be superior in inducing antigen-specific immune responses with elevated production of IL-4 and IFN-γ compared with CS-CL MPs under *ex vivo* re-stimulation conditions. Moreover, the proliferative response of splenocytes from mice immunized with CS-NH_2_ MPs was also observed to be elevated compared with mice immunized with CS-CL MPs, indicating the importance of free amino groups in the activation of antigen-specific immune responses with the involvement of the complement system. Even though the exact mechanism of complement activation by CS-NH_2_ MPs is not yet known, it has been postulated that free amino groups on CS-NH_2_ MPs interacting with C3b present in the plasma could have stabilized it and thus activated the complement system for a longer period. However, further studies are needed to verify this assumption [116].

### 7.2. Inulin

The role of inulin as an adjuvant has also been explored for activating the complement system. Inulin is a carbohydrate, which occurs in various forms of structural isomers named alpha, beta and gamma. Importantly, gamma inulin is more potent among the three forms in activating the complement system [117]. An intraperitoneal injection of gamma inulin induced infiltration of peritoneal neutrophils and lymphocytes by activating the complement system. The mechanism by which gamma inulin activates the complement system could be due to the enhanced deposition of C3 on APCs such as macrophages, leading to the activation of T cells [117,118]. However, detailed studies are needed to evaluate the role of inulin in activating the complement system.

### 7.3. Beta-glucans

Beta-glucans as adjuvants are well known to induce adaptive immunity by activating the complement system. Beta-glucans are the natural polysaccharides present in the cell walls of certain pathogens. Glucans activate the complement system by interacting with the cell surface complement receptor, CR3, present in macrophages, NK cells and B and T lymphocytes [119]. A well-known beta-glucan ‘Zymosan’ derived from *Saccharomyces cerevisiae,* activates the alternative complement pathway by interacting with macrophages. Interaction of zymosan with macrophages induces Th1 responses by secreting cytokines like IFN-γ, TNF-α, IL−2 and IL−8. Moreover, zymosan’s potential as an adjuvant to induce a strong humoral immune response by activating the complement system has been explored in an HIV-1 DNA vaccination study. In this study, elevated levels of antibody responses and HIV-specific delayed-type hypersensitivity were observed in mice immunized with HIV-1-specific DNA vaccine (pCMV160IIIB) along with zymosan compared with pCMV160IIIB alone. This superiority in the immune responses was explained by the activation of macrophages and dendritic cells by complement components [120]. A recent clinical trial evaluating the immunomodulatory effects of Reishi-derived β-glucan in healthy adults demonstrated a significant enhancement in immune cell populations, including CD3^+^, CD4^+^ and CD8^+^ T-lymphocytes, as well as natural killer cells. Additionally, the intervention group exhibited improvements in serum IgA levels and NK cell cytotoxicity compared with the placebo group. These findings highlight β-glucan’s potential in enhancing the immune responses, suggesting its role in bolstering defence against infections as an immunomodulatory agent [125]. Further studies on granular β-1,3-glucan (GPG) from *Ganoderma lucidum* spores reinforce this adjuvant potential, with GPG reversing chemotherapy-induced immunosuppression. GPG inhibits myeloid-derived suppressor cell differentiation through the Dectin-1 pathway, thereby increasing pro-inflammatory markers like MHC-II and CD86, while reducing immunosuppressive factors, showcasing its potential as an adjuvant and regulator in tumour microenvironments [126].

### 7.4. Alum

Aluminium hydroxide is the most common and approved adjuvant for human vaccines, which is known to induce adaptive immunity. The involvement of alum in the activation of all three complement pathways has been confirmed in a study in which aluminium hydroxide was treated with human serum, followed by a precipitation step [121]. Production of C3a and C5a anaphylatoxins, formation of the MAC and deposition of C3 cleavage components were observed in the precipitates. Aluminium hydroxide-mediated complement activation is a time-dependent process that can be inhibited by chelators like ethylenediaminetetraacetic acid (EDTA) [121]. Building on alum’s established role in immune response activation, a study evaluating the immune responses induced by alum and an oil-in-water emulsion adjuvant (MF59) in a modified RV144 HIV vaccine regimen provided further insights into the effectiveness of alum as an adjuvant. Both alum and MF59 induced similar levels of vaccine-specific IgG antibodies, suggesting that alum can effectively enhance immune responses after HIV vaccination, comparable with the more commonly used MF59 adjuvant. Notably, the alum’s contribution to eliciting IgA responses was also comparable with MF59. This finding suggests that the choice of an adjuvant may not be critical in certain vaccine formulations, like ALVAC-HIV+gp120. However, this study has limitations in clarifying the dose–response relationship because of the relatively small sample size used per group and the absence of higher gp120 doses [127]. Recent studies on novel adjuvant formulations continue to reinforce alum’s pivotal role in enhancing immune responses. For instance, a trial using toll-like receptor (TLR)7/8 signalling adjuvant, 3M-052-AF, combined with alum, demonstrated robust antibody production and T-cell activation in response to trimeric HIV vaccines. This combination elicited a broad array of neutralizing antibodies and induced a strong immune response, supporting alum’s continued relevance in promoting vaccine efficacy [128].

### 7.5. Nanoparticles

Synthetic particulates are also able to activate the complement system. Complement proteins that function like opsonins (C3b, C4b and C1q) are adsorbed on NP surfaces and provide signals to the phagocytic cells to induce the clearance of nanoparticles from the bloodstream. These proteins form a corona on the particle’s surface, which increases in proportion to the increased hydrophobicity of the particle’s surface. In this way, NPs can activate the complement system more robustly. The extent of activation depends on particle size and surface chemistry. For instance, NPs with different lipid-attached gadolinium chelates on the particle surface rapidly activated the complement system through the classical pathway, and the activation was mainly dependent on the chemistry of lipid-attached chelates and surface charges [129]. In another study, two different types of NPs, polymethoxylated (CH_3_O-NPs) and polyhydroxylated (OH-NPs) NPs, were studied for activating the complement system. Hydroxylated NPs induced much higher C3a levels compared with polymethoxylated particles. Hydroxylated NPs were also able to target lymph nodes and promote DC maturation and activation of CD8^+^ T-cell memory compared with the same-sized methoxylated NPs [122]. However, comprehensive studies are needed to explore the detailed role of NPs in activating the complement system. Recent developments in NP-based therapies also highlight their potential for immune modulation. For instance, NPs activating the NLR family pyrin domain containing 3 (NLRP3) inflammasomes, a crucial pathway in innate immune responses, can significantly enhance immunogenicity. A polymeric nano-vaccine platform, consisting of a poly(orthoester) scaffold with a TLR7/8 agonist and an endosomal escape peptide, was developed to induce lysosomal rupture and trigger NLRP3 inflammasome activation. These NPs, around 50 nm in size, co-delivered neoantigens to antigen-presenting cells, leading to strong antigen-specific CD8^+^ T-cell responses, including IFN-γ and granzyme B secretion. Moreover, when combined with immune checkpoint blockade therapy, they elicited potent anti-tumour immune responses in various tumour models [130,131].

### 7.6. Flagellin

The role of flagellin in activating the complement system for the induction of immune responses has been studied extensively. The well-known vaccinia virus antigens L1R and B5R are immunogenic, but they are toxic in their native forms. Their recombinant forms are also poorly immunogenic. Flagellin as an adjuvant has been studied with these vaccinia virus antigens in fusion forms like flagellin-L1R and flagellin-B5R [123]. Immunization of these fusion proteins in mice confers significant protection against the vaccinia virus challenge by activating the complement system. Depletion of complement components using cobra venom factor in vaccinated mice confirmed the involvement of the complement system in protecting the animals [123]. Recent studies highlighted the role of pathogen-associated molecular patterns (PAMPs), such as flagellin (FLA), in activating TLR-mediated immune responses. For example, in an *in vitro* model using human-induced pluripotent stem cell-derived cardiomyocytes (hiPSC-CMs), higher doses of flagellin led to increased levels of inflammation-associated cytokines like TNF-α, as well as the activation of downstream signalling pathways, including caspase-8. These responses were linked to enhanced TLR5 expression, demonstrating FLA-induced innate immune processes [132]. Similarly, flagellin was shown to induce a robust innate immune response in human sinonasal epithelial cells (HSNECs). In this study, *P. aeruginosa*-derived flagellin triggered the secretion of pro-inflammatory cytokines like TNF-α, IL−1β and IL-17 and activated the IL-17C/IL-23 axis. Moreover, in patients with chronic rhinosinusitis (CRS) , HSNECs exhibited a dysregulated immune response to flagellin, marked by elevated TNF-α and reduced IL-6 secretion. These findings underline the role of flagellin in promoting inflammatory pathways and suggest that modulating immune responses via flagellin could offer potential therapeutic strategies for CRS [133].

### 7.7. Toll-like receptor agonists

Both the complement system and TLRs are critical parts of the innate immune system. Many microbial products act as activators of both forms of innate immunity. For instance, LPS, an agonist for TLR4, is also a well-known activator of the complement system [134]. Similarly, yeast cell wall component, zymosan, activating the alternative complement pathway, can also act as a ligand for TLR2/6 [135] and CpG oligonucleotide, a ligand for TLR9 [136], also activates the alternative complement pathway. Recently, interactions between complement and TLR pathways were studied by Zhang *et al.* [137] using LPS in decay-accelerating factor-deficient (DAF^−/−^) mice. Elevated expression of pro-inflammatory markers was observed after LPS injection compared with naive mice, as evidenced by an increased IL-6 mRNA synthesis. Similar results were observed when wild-type mice were treated with cobra venom factor and TLR ligands. Production of TLR-induced inflammatory cytokines was regulated through complement receptors, C3aR and C5aR [137]. In line with these findings, another study has also further highlighted the rapid activation of β2-integrins observed after TLR2 and TLR5 ligation, which enhanced leukocyte adhesion to endothelial cells, a key process in immune responses. This mechanism involves critical signalling pathways, including Rap1 GTPase, Rac1 and NADPH oxidase-2-dependent reactive oxygen species production, underscoring the importance of TLRs in regulating acute leukocyte infiltration [138]. Furthermore, TLR10, the most recently identified human TLR, plays a unique role in immune regulation by suppressing inflammation. Unlike other TLRs, TLR10 inhibits the activation of pro-inflammatory pathways by competing with stimulatory TLRs, through dimerization with TLR1, TLR2 and TLR6 and activating PI3K/Akt signalling to induce IL-1Ra production. These mechanisms position TLR10 as a potential modulator of immune responses, providing new opportunities for therapeutic immune regulation [139].

## 8. Conclusion

The complement system is an important component of our immune response to fight against pathogenic microbes. Under certain circumstances, complement components also contribute to triggering inflammatory events. Especially, deficiency in certain complement components leads to the initiation of several inflammatory syndromes, including autoimmune and neurological disorders. Activation of complement components was demonstrated in different preclinical preventive and therapeutic vaccination studies against different infectious and non-infectious diseases. To further enhance the immune responses involving complement activation, different natural and synthetic components are being explored as adjuvants. Although these studies have shown promising results at the preclinical level, evaluating them in clinical trials is needed to prove their efficacy and safety in humans.

## Data Availability

This article has no additional data.
